# Clinical outcomes of tirzepatide in a real-world follow-up cohort of patients with type 2 diabetes

**DOI:** 10.17925/EE.2026.22.2.1

**Published:** 2026-07-02

**Authors:** Thein M Tun, Muhammad Sabir, Hasanain Alismael, Alice Gatenby, Theint T Su, Lucy Walters, Timothy Green, Stephen C Bain, Richard Chudleigh, Rajesh Peter, Jeffrey W Stephens, Thinzar Min

**Affiliations:** 1. Diabetes Department, Morriston Hospital, Swansea Bay UHB, Swansea, UK; 2. Diabetes Department, Neath Port Talbot Hospital, Swansea Bay UHB, Swansea, UK; 3. Diabetes Department, Singleton Hospital, Swansea Bay UHB, Swansea, UK; 4. Diabetes Research Group, Swansea University Medical School, Swansea, UK

**Keywords:** Cohort study, glycemic control, incretin-based therapy, real-world evidence, secondary care, tirzepatide, type 2 diabetes, weight loss

## Abstract

**Introduction:**

Tirzepatide, a dual glucose-dependent insulinotropic polypeptide and glucagon-like peptide-1 receptor agonist (GLP-1RA), has demonstrated major metabolic benefits in clinical trials, but real-world evidence from secondary care remains limited. This study evaluated the metabolic impact, treatment persistence and subgroup differences in response to tirzepatide among adults with type 2 diabetes (T2D) managed in specialist services.

**Methods:**

This retrospective observational study included adults with T2D initiated on tirzepatide across three secondary-care diabetes centers between July 2024 and July 2025. Individuals with at least one follow-up visit were included. Outcomes assessed included glycated hemoglobin (HbA1c), weight, body mass index (BMI), blood pressure, lipid profile, alanine aminotransferase (ALT) and total daily insulin dose. Subgroup analyses examined the effects of prior GLP-1RA use, baseline HbA1c and baseline BMI using analysis of covariance models.

**Results:**

A total of 295 patients were included (mean age: 58.7 ± 11.7 years; 51.9% female), with a median follow-up of 6.7 months. At follow-up, the mean prescribed dose was 5 mg. Mean HbA1c decreased by 17.7 mmol/mol and body weight by 5.9 kg (both p<0.001). The proportion achieving HbA1c <58 mmol/mol rose from 15.3% to 47.5% (χ²=74.46; p<0.001). Weight-loss thresholds of >5%, >10%, >15% and >20% were achieved by 46.5%, 20.5%, 8.5% and 4.6% of participants, respectively. Insulin-treated individuals reduced their daily insulin dose by 13.5 units (p=0.001). Improvements were also observed in blood pressure, triglycerides, low-density lipoprotein cholesterol and ALT. Subgroup analyses showed broadly consistent benefits; GLP-1RA-naive status was associated with lower adjusted follow-up HbA1c and weight, while higher baseline BMI was associated with higher adjusted follow-up HbA1c.

**Conclusion:**

In routine secondary care, tirzepatide produced clinically meaningful improvements in glycemia, weight and cardiometabolic risk markers, with nearly half achieving HbA1c <58 mmol/mol. Benefits were consistent across key clinical subgroups, supporting its effectiveness in real-world practice.

Type 2 diabetes (T2D) is a progressive metabolic condition associated with obesity, cardiovascular disease and substantial morbidity and mortality.^1^ Despite the availability of multiple glucose-lowering therapies, a significant proportion of individuals with T2D fail to achieve recommended glycemic targets, particularly those with long disease duration, obesity and insulin resistance.^2,3^ In parallel, excess adiposity remains a major driver of insulin resistance and cardiometabolic risk, highlighting the need for therapies that can address both glycemic control and weight management.^4^

Glucagon-like peptide-1 receptor agonists (GLP-1RAs) have demonstrated benefits in glycemic control, weight reduction and cardiovascular outcomes, and are widely recommended in contemporary diabetes guidelines.^5,6^ However, responses to GLP-1RAs are heterogeneous, weight loss is often modest in routine practice, and therapeutic inertia or intolerance may limit long-term effectiveness.^7,8^ There, therefore, remains a clinical need for more effective and durable therapies for individuals with T2D, particularly those with obesity and inadequate glycemic control.

Tirzepatide is a novel dual glucose-dependent insulinotropic polypeptide and GLP-1RA that has demonstrated superior glycemic and weight-lowering efficacy compared with established GLP-1RAs in the SURPASS randomized controlled trial program.^9–14^ Across these trials, tirzepatide produced substantial reductions in glycated hemoglobin (HbA1c), body weight and cardiometabolic risk markers, with a safety profile consistent with incretin-based therapies.^9–12^ These findings have positioned tirzepatide as a potent therapeutic option for people with T2D, including those previously treated with GLP-1RAs or insulin.

However, randomized controlled trials are conducted in highly selected populations under tightly controlled conditions, which may limit generalizability to routine clinical practice. Patients managed in secondary care often have more advanced disease, higher baseline HbA1c, greater obesity and complex treatment regimens that may influence real-world effectiveness. Furthermore, the impact of prior GLP-1RA exposure, baseline glycemic control and degree of obesity on response to tirzepatide in routine practice remains incompletely characterized. Real-world evidence is therefore essential to complement trial data and to inform clinicians about expected outcomes in everyday settings.

In the UK, tirzepatide has been rapidly adopted into secondary care diabetes services following regulatory approval, including use in individuals with obesity, insulin-treated diabetes and those switching from other GLP-1RAs due to suboptimal response or medication shortages.^15,16^ This has created a timely opportunity to evaluate early real-world outcomes and to explore treatment responses across clinically relevant subgroups.

The aim of this study was to evaluate the real-world clinical effectiveness of tirzepatide in a secondary care T2D cohort, focusing on changes in glycemic control, weight, cardiometabolic risk markers and insulin requirements. In addition, we examined treatment response according to prior GLP-1 RA exposure, baseline glycemic control and baseline body mass index (BMI), to better understand which patient groups may derive the greatest benefit from tirzepatide therapy in routine clinical practice.

## Methods

### Study design and population

All adults with T2D who were consecutively initiated on tirzepatide between July 2024 and July 2025 across three hospital sites were identified through a local electronic clinical database. Of 329 eligible individuals, 295 patients who had attended at least one follow-up visit were included in the final analysis. The 34 individuals without follow-up were predominantly initiated near the end of the study period, leaving insufficient time for review. Their baseline age, HbA1c and BMI were comparable to those included in the cohort.

### Data collection

Demographic characteristics and background glucose-lowering therapies were recorded. Anthropometric measurements included body weight, height and BMI. Routinely collected clinical parameters included systolic blood pressure (SBP) and diastolic blood pressure (DBP). Biochemical variables included HbA1c, lipid profile (total cholesterol, triglycerides, low-density lipoprotein [LDL] and high-density lipoprotein [HDL] cholesterol) and alanine aminotransferase (ALT). Reasons for initiating or switching to tirzepatide were extracted from clinic correspondence.

### Outcomes

Changes in routinely collected clinical and biochemical variables from baseline to follow-up were evaluated. In addition to analyses of the overall cohort, subgroup analyses were performed in three clinically relevant strata:

- Prior GLP-1RA exposure (GLP-1RA-naive versus prior GLP-1RA users) • Baseline glycemic control, defined using an HbA1c threshold of 75 mmol/mol (9.0%)
- Baseline BMI, stratified using a cut-off of 35.0 kg/m².

The HbA1c threshold of 75 mmol/mol was selected in line with National Institute for Health and Care Excellence (NICE) guidance, which recommends consideration of insulin therapy above this level and reflects a threshold at which clinicians would typically escalate treatment beyond oral agents.^17^ The BMI threshold of 35.0 kg/m² was used in accordance with NICE criteria for GLP-1RA initiation.^18^

### Compliance with ethics guidelines

Formal research ethics committee approval was not required, as this analysis was conducted as a service evaluation examining the effects of tirzepatide following its introduction into routine clinical practice within our local population.

### Statistical analysis

Statistical analyses were performed using SPSS version 32 (IBM Corp., Armonk, NY, USA). Continuous variables are presented as mean ± standard deviation. The Shapiro–Wilk test was used to assess data normality. Changes in clinical and biochemical parameters from baseline to follow-up were analyzed using paired *t*-tests. The Kruskal–Wallis test was used to compare glycemic and weight parameters across the dosage range of tirzepatide and follow-up duration. Baseline characteristics between subgroups were compared using independent samples *t*-tests. The effects of tirzepatide on HbA1c, weight and BMI in subgroup analyses were assessed using analysis of covariance (ANCOVA), adjusting for age, sex, baseline HbA1c, baseline weight, baseline BMI, duration of follow-up and tirzepatide dose at follow-up as appropriate. A two-sided p-value <0.05 was considered statistically significant.

## Results

### Baseline characteristics

The final cohort (n=295) had a mean age of 58.7 ± 11.7 years, with a slight female predominance (51.9%). Mean baseline HbA1c was 78.8 ± 19.5 mmol/mol, mean body weight was 112.8 ± 25.7 kg, and mean BMI was 39.5 ± 8.4 kg/m². Only 15.7% of participants had achieved an HbA1c target of <58 mmol/mol at baseline.

**Table 1:** Baseline glucose-lowering therapy

Medications (n=295)	n	%
Insulin	164	55.6
Metformin	204	69.2
SGLT2i	154	52.2
DPP-4	11	3.7
Sulfonylurea	55	18.6
GLP-1RA	175	59.3
• Dulaglutide	59	20
• Liraglutide	16	5.4
• Oral semaglutide	41	13.9
• Injectable semaglutide	44	14.9
• Xultophy	15	5.1

*DPP-4 = dipeptidyl peptidase 4; GLP-1RA = glucagon-like peptide-1 receptor agonist; SGLT2i = sodium/glucose cotransporter 2 inhibitor.*

Prior to initiation of tirzepatide, 55.6% of patients were treated with insulin, and 59.3% were receiving GLP-1RA therapy. Background glucose-lowering therapies are summarized in *Table 1*. Reasons for initiating or switching to tirzepatide included inadequate glycemic control, shortages of prescribed GLP-1RA agents and the need for additional weight loss. The median duration of follow-up was 6.7 months, providing adequate exposure to detect changes in cardiometabolic risk markers.

### Changes in cardiometabolic risk markers

Clinically meaningful improvements were observed across multiple metabolic parameters following initiation of tirzepatide (*Table 2*). Mean HbA1c decreased by 17.7 mmol/mol at follow-up (p<0.001). The proportion of patients achieving HbA1c <58 mmol/mol increased from 15.3% at baseline to 47.5% at follow-up (χ²=74.46; p<0.001). Similar improvements were observed at more stringent HbA1c thresholds (*Figure 1*). Mean body weight decreased by 5.9 kg (p<0.001), with a corresponding mean BMI reduction of 1.8 kg/m² (p<0.001). Clinically significant weight loss of >5%, >10%, >15% and >20% was achieved by 46.5%, 20.5%, 8.5% and 4.6% of participants, respectively. Significant reductions were also observed in SBP (-7.5 mmHg; p<0.001), DBP (-3.4 mmHg), LDL cholesterol, triglycerides and ALT (-4.5 U/L; p=0.004). No significant changes were observed in total cholesterol or HDL cholesterol.

**Table 2:** Changes in cardiometabolic risk factors

Cardiometabolic risk factor	n	Baseline	Follow-up	Mean change (95% CI)	p-value
HbA1c (mmol/mol)	281	78.8 ± 19.5	61.1 ± 17.4	-17.7 (-19.8, -15.5)	<0.001
Weight (kg)	259	112.8 ± 25.7	106.9 ± 25.0	-5.9 (-7.2, -4.6)	<0.001
BMI (kg/m^2^)	258	39.5 ± 8.5	37.8 ± 10.5	-1.8 (-0.8, -2.7)	0.001
SBP (mmHg)	191	138.5 ± 17.6	131.0 ± 19.2	-7.5 (-10.3, -4.7)	<0.001
DBP (mmHg)	191	80.9 ± 9.9	77.7 ± 8.4	-3.2 (-4.6, -1.9)	<0.001
TDD (units)	122	81.3 ± 54.9	67.8 ± 51.7	-13.5 (-21.3, -5.8)	0.001
TC (mmol/L)	233	4.2 ± 1.1	4.0 ± 2.2	-0.3 (-0.5, 0.01)	0.064
HDL (mmol/L)	236	1.1 ± 0.3	1.1 ± 0.3	-0.0 (-0.04, 0.01)	0.279
LDL (mmol/L)	205	2.0 ± 0.8	1.8 ± 0.8	-0.2 (-0.2, -0.1)	<0.001
TG (mmol/L)	233	2.8 ± 2.1	2.3 ± 2.8	-0.5 (-0.9, -0.1)	0.015
ALT (IU/L)	154	30.8 ± 20.9	26.2 ± 13.6	-4.5 (-7.6, -1.5)	0.004

*Data presented as the mean ± SD. Mean change is follow-up minus baseline; negative values indicate improvement. The p-values are from paired* t*-tests.*

*ALT = alanine aminotransferase; BMI = body mass index; CI = confidence interval; DBP = diastolic blood pressure; HbA1c = glycated hemoglobin; HDL = high-density lipoprotein; LDL = low-density lipoprotein; SBP = systolic blood pressure; SD = standard deviation; TC = total cholesterol; TDD = total daily insulin dose; TG = triglyceride.*

Among patients treated with insulin (n=122), mean total daily insulin dose decreased from 81.3 ± 54.9 units to 67.8 ± 51.7 units, representing a reduction of 13.5 units/day (p=0.001).

At follow-up, the most commonly prescribed dose was 5 mg (n=149; 50%), followed by 7.5 mg (16.3%). Patients receiving 5 mg demonstrated mean reductions of -20.2 mmol/mol in HbA1c (95% confidence interval [CI]: -23.1 to -17.4) and -5.6 kg in body weight (95% CI: -7.5 to -3.8). The 10 mg dose group (n=20) showed larger reductions in HbA1c (-23.2 mmol/mol; 95% CI: -32.1 to -14.2) and weight (-9.9 kg; 95% CI: -15.1 to -4.6), though estimates were less precise due to the smaller sample size. Higher dose categories were infrequent (12.5 mg, n=3; 15 mg, n=1), precluding meaningful analysis (*Tables 3 and 4*).

When stratified by duration of follow-up, HbA1c reductions were evident within the first 3 months (mean: -15.6 mmol/mol) and were greatest between 3 and 6 months (mean: -21.4 mmol/mol). Weight loss increased incrementally with longer follow-up, reaching -7.7 kg (95% CI: -11.0 to -4.5) in the 12- to 18-month band (*Tables 3 and 4*).

## Subgroup analyses

### Prior glucagon-like peptide-1 receptor agonist exposure

There were no significant differences in baseline characteristics between patients who are GLP-1RA-naive and those previously treated with GLP-1RA therapy (*Table 5*). Significant improvements in glycemic and body weight measures were observed in both groups. After adjusting for age, sex, baseline HbA1c, baseline weight, baseline BMI, follow-up duration and tirzepatide dose in ANCOVA models, GLP-1RA-naive status was significantly associated with a greater HbA1c reduction, with adjusted follow-up HbA1c values of 59.1 versus 62.7 mmol/mol (*F*=4.73; p=0.031). GLP-1RA-naive status was also significantly associated with lower adjusted follow-up weight (105.1 versus 108.2 kg; *F*=6.15; p=0.015). However, the association with follow-up BMI did not reach statistical significance (*F*=3.63; p=0.059).

### Baseline glycemic control

Participants with baseline HbA1c ≥75 mmol/mol experienced a greater unadjusted reduction in HbA1c (-25.2 mmol/mol) compared with those with HbA1c <75 mmol/mol (-8.4 mmol/mol) (*Table 6*). However, after adjustment in ANCOVA models, baseline glycemic status was not independently associated with follow-up HbA1c (p=0.773), weight (p=0.721) or BMI (p=0.399).

### Baseline body mass index

Glycemic and weight outcomes by baseline BMI are shown in *Table 7*. Among 259 participants with BMI data, baseline BMI category was associated with follow-up HbA1c (p=0.009), with higher adjusted HbA1c in participants with BMI ≥35 kg/m² (63.3 versus 56.7 mmol/mol). Baseline BMI category was not associated with follow-up weight (p=0.752) and showed a borderline association with follow-up BMI (p=0.054).

### Treatment persistence and tolerability

Treatment continuation rates were high, with approximately 89% of patients with documented follow-up remaining on tirzepatide. Thirty-three patients (11%) discontinued tirzepatide. Most individuals who discontinued tirzepatide did so because of gastrointestinal intolerance (n=14; 44%). Other reasons for discontinuation included non-response (n=6), diabetic ketoacidosis (n=2), pregnancy (n=1), cancer (n=2) and death (n=2).

**Figure 1:**
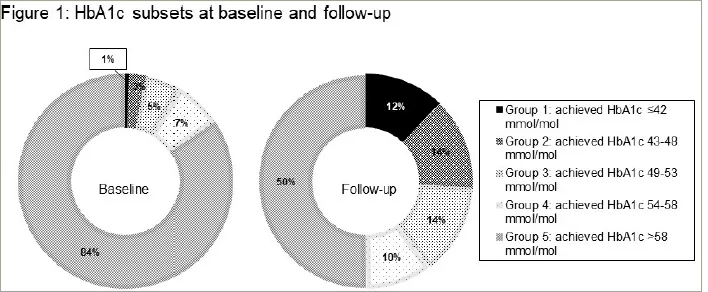
Glycated hemoglobin subsets at baseline and follow-up

*HbA1c = glycated hemoglobin.*

## Discussion

In this real-world secondary care cohort of people with T2D, initiation of tirzepatide was associated with substantial improvements in glycemic control, body weight, cardiometabolic risk markers and insulin requirements over a median follow-up of 6.7 months. These findings are consistent with, and complementary to, the efficacy demonstrated in the phase III SURPASS program, while providing important context regarding the effectiveness of tirzepatide in a more complex, treatment-experienced population typical of secondary care practice.

In the SURPASS trials, tirzepatide produced mean HbA1c reductions of approximately -1.8% to -2.6% (-20 to -28 mmol/mol) and weight reductions of -7 to -12 kg, depending on dose and trial population.^11^ Of note, in the SURPASS Cardiovascular Outcome Trial (SURPASS-CVOT) study, tirzepatide achieved an additional ~0.5% HbA1c reduction and ~5 kg weight reduction when compared with dulaglutide.^12^ In our cohort, the mean HbA1c reduction of -17.7 mmol/mol and mean weight loss of -5.9 kg are directionally consistent but more modest in magnitude.

This difference is unsurprising given that the majority of our cohort were previous GLP-1RA users who had already achieved reasonable glycemic control before initiation of tirzepatide, leaving less scope for further HbA1c improvement. Moreover, most patients in our cohort were maintained on 5 mg at follow-up, in contrast to higher dosage regimes more commonly represented in the SURPASS trials.^9–12^ Despite most patients receiving 5 mg, approximately 50% of patients achieved an HbA1c of <58 mmol/mol (a commonly used threshold when considering treatment intensification), and a quarter achieved HbA1c <48 mmol/mol. In the SURPASS program, the proportion achieving normoglycemia was higher, with 37% achieving HbA1c <5.7%, 44% achieving HbA1c 5.7–6.5% and 19% remaining >6.5%.^11^

Notably, glycemic improvements were evident within the first 3 months and were greatest between 3 and 6 months, mirroring the early and sustained HbA1c reductions observed in SURPASS studies. Similarly, weight loss increased with longer follow-up duration, supporting the progressive weight-lowering effect of tirzepatide described in trials and post-hoc analyses.^19,20^ The observed dose–response relationship, with numerically greater HbA1c and weight reductions at higher tirzepatide doses, also aligns with trial data, although interpretation in our cohort is limited by smaller numbers at higher doses.

**Table 3:** Glycemic outcomes by tirzepatide dose at follow-up and follow-up duration

(a) dose at FU (mg)	n	FU duration, months	HbA1c baseline, mmol/mol	HbA1c FU, mmol/mol	HbA1c, mmol/mol (95% CI)	p (∆HbA1c)
2.5	21	8.0 (4.7)	86.2 (22.5)	69.5 (17.1)	-16.7 (-28.2, -5.2)	0.022
5	149	6.4 (4.1)	78.0 (18.6)	57.7 (15.8)	-20.2 (-23.1, -17.4)	
7.5	45	10.7 (4.1)	75.6 (18.1)	64.9 (19.0)	-10.7 (-14.8, -6.6)	
10	20	10.7 (4.1)	82.6 (22.7)	59.5 (17.0)	-23.2 (-32.1, -14.2)	
12.5	3	12.4 (6.6)	81.3 (27.8)	60.3 (12.9)	-21.0 (-62.0, 20.0)	
15	1	15.2 (NA)	51.0 (NA)	33.0 (NA)	-18.0 (NA)	
**(b) FU duration (months)**	**n**	**HbA1c baseline, mmol/mol**	**HbA1c FU, mmol/mol**	**∆HbA1c, mmol/mol (95% CI)**	**p (∆HbA1c)**	
0–3	44	73.8 (17.1)	58.2 (16.8)	-15.6 (-19.5, -11.7)	0.444	
>3–6–6	60	78.5 (19.0)	57.2 (13.1)	-21.4 (-25.9, -16.8)		
>6–9–9	41	76.5 (22.5)	57.7 (18.2)	-18.8 (-24.8, -12.8)		
>9–12–12	41	81.4 (20.2)	61.8 (17.0)	-19.6 (-25.9, -13.3)		
>12–18–18	53	81.7 (18.4)	66.1 (18.9)	-15.7 (-21.2, -10.1)		
>18–24–24	1	67.0 (NA)	42.0 (NA)	-25.0 (NA)		

*Data presented as the mean ± SD unless otherwise stated. Change (∆) is follow-up minus baseline; negative values indicate improvement. ∆HbA1c is presented as mean (95% CI). The p-values are from Kruskal–Wallis tests comparing ∆HbA1c across strata (excluding strata with n<2 from the test). (a) glycemic outcomes by dose at FU (p [∆HbA1c]=0.022) and (b) glycemic outcomes by FU duration (p [∆HbA1c]=0.444)*

*CI = confidence interval; FU = follow-up; HbA1c = glycated hemoglobin; NA = not applicable; SD = standard deviation; ∆HbA1c = mean change in HbA1c.*

**Table 4:** Weight outcomes by tirzepatide dose at follow-up and follow-up duration

(a) Dose at FU (mg)	n	FU duration, months	Weight baseline, kg	Weight FU, kg	∆weight, kg (95% CI)	%∆Weight (mean ± SD)	p (∆weight)
2.5	21	8.0 (4.7)	103.5 (17.8)	100.8 (19.6)	-2.7 (-5.3, -0.1)	2.8 (5.2)	0.449
5	149	6.4 (4.1)	111.7 (25.1)	106.1 (24.7)	-5.6 (-7.5, -3.8)	-4.6 (11.3)	
7.5	45	10.7 (4.1)	118.4 (24.3)	112.5 (25.5)	-6.0 (-8.9, -3.1)	-5.1 (7.9)	
10	20	10.4 (3.4)	122.4 (37.9)	112.5 (35.4)	-9.9 (-15.1, -4.6)	-7.6 (8.6)	
12.5	3	12.4 (6.6)	105.7 (16.0)	104.1 (23.5)	-1.6 (-36.3, 33.1)	-2.0 (14.1)	
15	1	15.2 (NA)	181.0 (NA)	130.8 (NA)	-50.2 (NA)	-27.7 (NA)	
**(b) FU duration (months)**	**n**	**Weight baseline, kg**	**Weight FU, kg**	**∆Weight, kg (95% CI)**	**%∆Weight (mean ± SD)**	**p (∆weight)**	
0–3	44	114.1 (28.3)	110.6 (27.8)	-3.5 (-7.2, 0.2)	-2.7 (10.5)	0.728	
>3–6–6	60	113.3 (27.2)	106.6 (25.8)	-6.7 (-9.8, -3.6)	-5.1 (13.7)		
>6–9–9	41	111.5 (26.9)	105.9 (26.5)	-5.6 (-8.3, -2.8)	-4.9 (7.0)		
>9–12–12	41	115.8 (27.8)	110.2 (28.1)	-5.6 (-8.5, -2.7)	-4.8 (8.0)		
>12–18–18	53	112.5 (21.9)	104.8 (20.4)	-7.7 (-11.0, -4.5)	-6.5 (8.9)		
>18–24–24	1	102.0 (NA)	101.0 (NA)	-1.0 (NA)	-1.0 (NA)		

*p (∆weight) = 0.449. Values are mean (SD) unless otherwise stated. Change (∆) is follow-up minus baseline; negative values indicate improvement. ∆Weight is presented as mean (95% CI). The p-values are from Kruskal–Wallis tests comparing ∆Weight across strata (excluding strata with n<2 from the test). (a) weight outcomes by dose at follow-up and (b) weight outcomes by follow-up duration*

*C = confidence interval; FU = follow-up; NA = not applicable; SD = standard deviation.*

**Table 5:** Glycemic and weight outcomes by glucagon-like peptide-1 receptor agonist groups

Metabolic risk factor	GLP1-RA-naive group				Previous GLP-1RA users			
	Baseline	Follow-up	Mean change	p-value	Baseline	Follow-up	Mean change	p-value
HbA1c (mmol/mol)	80.4 ± 21.0	59.6 ± 18.7	-20.8	<0.001	77.8 ± 18.5	62.2 ± 16.5	-15.5	<0.001
Weight (kg)	114.3 ± 27.3	106.6 ± 26.8	-7.7	<0.001	111.9 ± 24.7	107.2 ± 23.9	-4.7	<0.001
BMI (kg/m^2^)	39.2 ± 8.2	36.5 ± 7.6	-2.7	<0.001	39.7 ± 8.7	38.6 ± 12.0	-1.1	0.172

*Data presented as the mean ± SD. Mean change is follow-up minus baseline; negative values indicate improvement. The p-values are from paired* t*-tests. BMI = body mass index; GLP1-RA = glucagon-like peptide-1 receptor agonist; HbA1c = glycated hemoglobin; SD = standard deviation.*

**Table 6:** Glycemic and weight outcomes by baseline glycemic status

Metabolic risk factor	Baseline HbA1c <75 mmol/L				Baseline HbA1c ≥75 mmol/L			
	Baseline	Follow-up	Mean change	p-value	Baseline	Follow-up	Mean change	p-value
HbA1c (mmol/mol)	61.3 ± 8.9	52.9 ± 14.4	-8.4	<0.001	93.1 ± 13.2	67.9 ± 16.9	-25.2	<0.001
Weight (kg)	117.4 ± 30.4	111.1 ± 28.3	-6.4	<0.001	109.1 ± 20.7	103.6 ± 21.6	-5.5	<0.001
BMI (kg/m^2^)	41.4 ± 10.0	39.1 ± 8.8	-2.3	<0.001	38.0 ± 6.7	36.7 ± 11.6	-1.3	0.130

*Data presented as the mean ± SD. Mean change is follow-up minus baseline; negative values indicate improvement. The p-values are from paired* t*-tests. BMI = body mass index; HbA1c = glycated hemoglobin; SD = standard deviation.*

**Table 7:** Glycemic and weight outcomes by baseline body mass index

Metabolic risk factor	Baseline BMI <35 kg/m^2^				Baseline BMI ≥35 kg/m^2^			
	Baseline	Follow-up	Mean change	p-value	Baseline	Follow-up	Mean change	p-value
HbA1c (mmol/mol)	81.0 ± 21.4	60.8 ± 17.9	-20.2	<0.001	77.9 ± 18.7	61.4 ± 17.4	-16.5	<0.001
Weight (kg)	94.6 ± 14.1	90.4 ± 14.2	-4.1	<0.001	121.3 ± 25.5	114.6 ± 25.3	-6.7	<0.001
BMI (kg/m^2^)	31.6 ± 2.8	30.3 ± 2.9	-1.4	<0.001	43.2 ± 7.7	41.3 ± 10.9	-1.9	0.008

*Data presented as the mean ± SD. Mean change is follow-up minus baseline; negative values indicate improvement. The p-values are from paired* t*-test. BMI = body mass index; HbA1c = glycated hemoglobin; SD = standard deviation.*

Reductions in both SBP and DBP were observed across the cohort and in all major subgroups. These changes, although modest, are clinically relevant given the high baseline cardiovascular risk in this population and in line with findings from clinical trials.^11,13^ Improvements in triglycerides and LDL cholesterol further support a favorable cardiometabolic profile associated with tirzepatide therapy, as demonstrated in clinical trials.^11,13^ The absence of significant change in HDL cholesterol is consistent with previous GLP-1RA real-world studies and likely reflects the short-to-medium follow-up duration.^21^

The observed reduction in ALT suggests a potential beneficial effect on hepatic steatosis or metabolic liver dysfunction, an effect previously reported with GLP-1RA therapy.^22,23^ While causality cannot be inferred from this study, these findings are encouraging and warrant further prospective evaluation.

Our findings are highly concordant with emerging real-world evidence. A recent systematic review and meta-analysis of 13 observational studies involving over 89,000 individuals reported a mean HbA1c reduction of -0.91% (-10 mmol/mol) and weight loss of -9.7 kg, with 47%, 23%, 9% and 4% of patients achieving ≥5%, ≥10%, ≥15% and ≥20% weight loss, respectively. The proportions achieving clinically meaningful weight loss in our cohort closely mirror these estimates, reinforcing the external validity of our findings.^24^ Real-world semaglutide studies in secondary care settings have similarly reported substantial improvements in glycemia and weight, though typically with more modest weight loss than observed for tirzepatide in comparative analyses.^21^

Large real-world cohort studies from the USA have demonstrated HbA1c reductions of approximately -1.0% and weight loss of -7 to -10 kg over 10–12 months, with greater responses in GLP-1RA-naive individuals compared with those switching from another GLP-1RA.^25^ In line with these observations, our adjusted analyses demonstrated that GLP-1RA-naive status was associated with a statistically significant – though clinically modest (~4 mmol/mol) – greater reduction in HbA1c. GLP-1RA-naive individuals also achieved significantly lower adjusted follow-up weight compared with prior GLP-1RA users, reinforcing the pattern of enhanced metabolic responsiveness in patients who are incretin-naive. Importantly, these data also suggest that tirzepatide provides additional glycemic benefit even in patients previously treated with GLP-1RA therapy, although weight responses remain partially attenuated in this group – findings that are clinically relevant in health systems where switching between incretin therapies is common.^26^

Although participants with higher baseline HbA1c experienced greater unadjusted glycemic reductions, baseline glycemic category was not independently associated with follow-up outcomes after adjustment, consistent with SURPASS analyses indicating benefits across a wide HbA1c spectrum. Participants with baseline BMI ≥35 kg/m² had higher adjusted HbA1c at follow-up, suggesting that severe obesity may attenuate glycemic response, despite substantial absolute benefits. Importantly, patients treated with insulin experienced a significant reduction in insulin dose, supporting the insulin-sparing effect of tirzepatide reported in SURPASS-5 and real-world cohorts.^13,27^

The predominance of the 5 mg dose in our cohort likely reflects several factors. First, dose escalation in routine clinical practice is often slower than in clinical trials, particularly in individuals prone to gastrointestinal intolerance, leading many patients to remain on lower doses for longer. Second, follow-up intensity in secondary care – typically less frequent than trial-mandated reviews – reduces opportunities for timely dose titration. Finally, local guidance and resource constraints, including limited primary-care capacity to oversee titration, further contributed to a more conservative approach to dose escalation in this cohort.

Although adverse events were not systematically recorded, treatment discontinuation was used as a pragmatic real-world proxy for tolerability. The relatively low discontinuation rate observed in our cohort – with gastrointestinal intolerance being the most common reason for cessation – is consistent with the known safety profile of incretin-based therapies and broadly comparable to findings from the SURPASS trials.^11^ In contrast, real-world persistence appears considerably lower in broader healthcare settings. A large US health system analysis, including both semaglutide (n=6,109) and tirzepatide (n=1,772), reported that more than half of patients discontinued treatment within the first year, encompassing both early (≤3 months) and late (3–12 months) discontinuation.^28^ Similarly, population-level US data demonstrated that among over 125,000 GLP-1RA initiators (including tirzepatide), 1-year discontinuation rates reached 46.5% in individuals with T2D and 64.8% in those without T2D, highlighting the extent of early cessation in routine practice.^29^ It is important to interpret the relatively low discontinuation rate in the context of the shorter follow-up period (median: 6.7 months) compared with the 12-month persistence estimates reported in larger health system datasets.

Estimates from comparative real-world analyses suggest tirzepatide discontinuation rates of approximately 18% at 12 months, though reported rates vary according to indication, access to specialist care and follow-up intensity. Importantly, practice context appears to play a substantial role: a Japanese secondary care cohort with structured fortnightly follow-up demonstrated an exceptionally low discontinuation rate of ~1.3%, with most cessations due to gastrointestinal adverse events – markedly lower than those reported in Western cohorts.^30^ Taken together, these data suggest that tirzepatide is generally acceptable in routine clinical practice – even among patients with severe obesity and complex comorbidity – and that specialist-led titration, proactive gastrointestinal symptom management and structured follow-up may help optimize persistence and narrow the gap between clinical trial efficacy and real-world outcomes.

## Strengths and limitations

Strengths of this study include the relatively large cohort, inclusion of patients previously treated and evaluation of clinically relevant subgroups. Limitations include the retrospective design, lack of a comparator group, variable follow-up duration and limited numbers at higher tirzepatide doses. Additionally, the lack of systematic adverse event reporting limits a detailed safety assessment. Longer follow-up and national-level data will be important to evaluate durability, tolerability and long-term outcomes.

## Conclusion

In this real-world audit, tirzepatide was associated with clinically meaningful improvements in glycemic control, body weight, insulin requirements and multiple cardiometabolic risk factors in adults with T2D and obesity managed in routine clinical practice. Benefits were observed across key subgroups, including individuals with severe hyperglycemia, prior exposure to GLP-1RA therapy and varying degrees of obesity, supporting the broad applicability of tirzepatide in complex metabolic disease. While absolute effect sizes are smaller than those observed in randomized trials, the magnitude of improvement remains substantial and highly relevant for real-world practice.
